# Suicidal Behavior in the Mediterranean Countries

**DOI:** 10.2174/1745017902016010093

**Published:** 2020-07-30

**Authors:** Mehmet Eskin

**Affiliations:** 1 Department of Psychology, College of Social Sciences and Humanities, Koç University, Rumelifeneri Yolu, 34450 Sariyer, Istanbul, Turkey

**Keywords:** Suicidal behavior, Mediterranean, Social indicators, Mental health, Suicidal mortality, Sucide rates

## Abstract

**Introduction:**

Suicidal behavior is a serious public health problem worldwide and shows large intersocietal variation. This study aimed at comparatively investigating the aspects of suicidal behavior in 22 countries surrounding the Mediterranean Sea.

**Methods:**

The study was conducted with official data retrieved from several sources. The suicidal mortality data were collected from World Health Organization’s data repository. Descriptive statistics, group comparison, correlational and regression statistical analyses were used to summarize the data.

**Results:**

The average age standardized suicide rates in the Mediterranean countries are lower than the world average. Except in Morocco, more men kill themselves than women. Suicide rates are lower in Mediterranean Muslim than in Mediterranean Christian countries. Slovenia, France and Croatia have the highest suicide mortality rates. Greatest percentages of suicidal ideation are seen in Croatia, Turkey and Slovenia and the greatest percentages of suicidal attempts are seen in Palestine, Cyprus, Greece and Slovenia. According to the results of the multiple regression analyses, the coefficient of human inequality index was associated with lower both-sex and male suicide rates. Greater percentages of people saying religion is unimportant in daily life in a country were found to be related to higher female suicide rates.

**Conclusion:**

The findings from the study have shown that the prevalence of suicidal deaths, thoughts and attempts vary between the Mediterranean countries. Lower suicide rates are observed in the Muslim Mediterranean nations than in the Judeo-Christian ones. However, the rates of suicide mortality in non-Arab Muslim nations being comparable to the rates in non-Muslim countries confirm the concerns over mis/underreporting of suicidal behavior in Arab Muslim countries due to religio-cultural stigma attached to suicide. The average suicidal mortality rates are lower in Mediterranean countries than the world average. Generally, more men than women kill themselves. Results from the multivariate analysis revealed that as the level of human inequality increases the rates for both-sex and male suicidal mortality decreases. Religion seem to be protective against female suicides. The study has also shown that more research is needed about suicidal behavior in the Mediterranean countries.

## 1 INTRODUCTION

Suicidal thoughts, plans, attempts and deaths that are collectively termed as suicidal behavior constitute a significant public health concern for all societies. Suicide causes many premature deaths and is a tragedy not only for the deceased but also for families, friends, neighbors and society. Suicide is a preventable condition. According to World Health Organization (WHO), 804,000 people died by suicide annually worldwide in 2012 and it is the second leading cause of death in the ages of 15-29. The annual global age-standardized suicide rate is 11.4 per 100, 000 population (15.0 for males and 8.0 for females) [1]. Seventy eight percent of all global suicides occur in low-middle income countries and the data indicate that there are 20 attempts for every suicide mortality [1]. This means globally 16,000,000 individuals per annum attempt to kill themselves. Taking thoughts, plans and attempts into account, suicidal phenomena are without a doubt significantly contributing to the burden on national health care systems [2]. For instance, the annual cost of only suicide and suicide attempts are estimated to be 93.5 billion dollars in the United States [3].

Suicide is a preventable condition and the World Health Organization has adopted a mental health action plan which aims to reduce suicide rates by 10% by 2020 [1]. The success of this plan depends on the scientific knowledge base at hand and the willingness of member states to engage in the prevention policies and actions based on the scientific evidence. Therefore, there is an urgent need for scientific knowledge on the causal factors involved in and the etiology of societal variation of suicidal behaviors. The knowledge about causal mechanisms involved in the onset and the maintenance of suicidal process is a prerequisite for a possibility of prevention and treatment.

Prevention of suicidal behavior requires early identification of persons at risk for suicide. The scientific evidence indicates that the prediction of suicidal persons is not better than chance [4] and there is a large etiological heterogeneity [5]. However, improved recognition of clinical, psychological, social and biological factors may help to identify those at risk for suicidal behavior for prevention and treatment [5]. The research has identified some risk factors for and protective factors against suicidal behavior. Knowledge about these factors may create an opportunity for both the prevention and treatment efforts.

Research has identified several factors that may constitute a risk for suicidal behavior. Accordingly, factors associated with suicidal behaviors include mental health problems [6-10], stressful life events [11-14], having a sexual minority status [15-17], previous suicidal behaviors [18-20], physical and sexual abuse [21, 22], substance and alcohol abuse [23-26] and so on. Strongest protective factors against suicidal behaviors include social support and connectedness [26-28], problem-solving skills [11, 29, 30] and to some extent religiosity [31-33].

Research in suicidology shows two invariant features of suicidal behavior. One is that suicidal behavior is a gender typed behavior. With some exceptions, scholarly work has often showed that women outnumber men in reports of suicidal ideation and attempts but more men than women kill themselves [34]. This is known as “gender paradox” [35, 36] and relates to gender culture. The paradox has often been explained through a reference to the choice of method for and intent involved in suicidal behavior [37-39]. The scientific investigations provide support for the view that men make use of more lethal methods for suicidal behavior than women that is in line with the cultural gender stereotypes [40]. The other invariant feature of suicidal behavior is that the prevalence rates present a large intersocietal variation [41]. The etiology of intersocietal variations in suicidal behavior are assumed to be the social and cultural factors [42-43].

The purpose of this paper is to undertake a comparative investigation of the aspects of suicidal behaviors in the Mediterranean countries. There are 22 states surrounding the Mediterranean Sea and they all have different social, cultural, levels of economic development, legal systems, religions, life styles and so on. The most distinctive features of the Mediterranean social fabric include its own diet, familial collectivism, a relaxed life style and emotional nature of its people. The Mediterranean basin is the cradle of civilization. The world’s most powerful empires flourished in the Mediterranean basin and is currently home for the three Abrahamic religions: Judaism, Christianity and Islam. The three Middle Eastern religions strongly disapprove suicide [33, 44].

## 2 MATERIALS AND METHODS

The data for the study were collected from several sources. The data reported in this study were as follows:

### 2.1 Suicidal Mortality Data

Age standardized suicide rates (per 100, 000 population) for the Mediterranean countries were collected from the World Health Organization’s suicide data repository [45].

### 2.2 Suicidal Ideation and Attempts

A literature search was done to locate empirical studies reporting the percentages of suicidal ideation and attempts through google scholar. To ensure comparability, studies exploring suicidal thoughts and attempts in student samples were included.

### 2.3 Mental Health and Alcohol Use

Estimates of percentages of country populations with depressive and anxiety disorders were taken from a WHO publication [46]. Total alcohol consumption per capita was taken from World Bank data repository [47]. It gives country specific liters of alcohol consumed per capita.

### 2.4 Social Indicators

Eight social indicators that may have a relevance for suicide were included in the study. Human development index for 2016; Coefficient of human inequality index for 2016 and Gender inequality index for 2016 were extracted from United Nations Development Program data repository [48]. The country Gross Domestic Product per capita (PPP) for 2016 and Unemployment rates (% unemployed) for 2016 were extracted from World Bank resources [47]. Percentages of people saying religion is unimportant in daily life based on Gallup Poll data was retrieved from RationalWiki [49]. Finally, country democracy index for the year 2017 was collected from the Economist’s Intelligence Unit web site [50].

### 2.5 Statistical Analysis

Descriptive statistics were used to calculate the average age standardized suicide rates and Mann-Whitney U test was used for group comparisons. Pearson product moment correlation coefficients were calculated to examine the bivariate associations of mental health and social indicators to suicidal mortality rates. Stepwise multiple regression analyses were performed to examine the independent predictors of suicide rates.

## 3 RESULTS

### 3.1 Suicidal Mortality

Age standardized suicide rates (per 100, 000 population) are presented in Table **1**. The table shows that Slovenia, France and Croatia have the highest, Montenegro, Turkey, Malta, Bosnia-Herzegovina, Spain, Albania, Italy, Libya and Israel have the medium high, and Cyprus, Egypt, Greece, Lebanon, Tunisia, Morocco and Syria have the lowest age standardized suicide rates.

The average age standardized suicide rates were 6.04 ( 3.09) for both genders and it was 9.29 (for males) and 2.95 (for females). Except for Morocco where more women killed themselves than men, more men killed themselves than women.

The average age standardized both-sex (Mean = 7.77±3.61 *versus* 4.62±1.68; Mann-Whitney Z = 1.98, p < 0.05) and male (Mean = 12.32±5.93 *versus* 6.81±3.07; Mann-Whitney Z = 2.13, p < 0.05) suicide rates were higher in Christian countries than in Muslim countries but the female suicide rates (Mean = 3.47±1.63 *versus* 2.52; Mann-Whitney Z = 1.37, p > 0.05) were similar in the two groups of countries.

### 3.2 Suicidal Ideation and Attempts

Table **2** presents the frequency of suicidal ideation and attempts in the Mediterranean countries. The table shows considerable variation in the rates of suicidal ideation and attempts. The highest percentages of suicidal ideation are observed in Croatia, Turkey and Slovenia, the lowest rates are seen in France, Spain and Lebanon. The highest suicide attempt rates are seen in Palestine, Cyprus, Greece and Slovenia but the lowest rates were in Spain, Italy, Lebanon and Morocco.

### 3.3 Social Indicators and Suicidal Mortality: Bivariate Analysis

The bivariate correlation coefficients between some social indicators and age standardized suicide rates are displayed in Table **3**. Human development index, democracy index, percentage of population saying religion is unimportant, GDP per capita and alcohol consumption per capita are positively associated with suicide mortality rates. Coefficient of human inequality and gender inequality indices are inversely related to suicide rates.

### 3.4 Social Indicators and Suicidal Mortality: Multivariate Analysis

According to the results of the stepwise multiple regression analyses, the coefficient of human inequality index was the only significant predictor of both sex [B = - 0.341, β = - 0.689, t = - 4.037, p < 0.005, % 95CI for B = - 0.518 to - 0.163] and male [B = - 0.603, β = - 0.717, t = - 4.358, p < 0.001, % 95CI for B = - 0.893 to - 0.312] suicide rates. This index accounted for 48% of the variance in both sex [F = 16.301, df = 19, p < 0.005] and 51% of the variance in male [F = 18.992, df = 19, p < 0.001] suicide rates. Percentage of population saying religion is unimportant was the only independent predictor of female suicide rates [B = 0.040, β = 0.599, t = 3.175, p < 0.01, % 95CI for B = 0.014 - 0.067] and it accounted for 36% of the variance in female suicide rates [F = 10.078, df = 19, p < 0.01].

## 4 DISCUSSION

The Mediterranean region is known for its relaxed lifestyle and turquoise beaches not to mention its renowned diet based on vegetables, fruit and yoghurt. However, the region is remembered lately most by refugee crisis and human tragedies resulting from sinking boats filled with migrants from poor countries heading to the rich European countries. Considering the relaxed life style under sunny days, it is hard to associate self-killing within the context of this background. Do people really think about killing, attempting to kill and actually killing themselves despite a relaxed lifestyle while so many try hard to survive against all odds? Accordingly, this study investigated the prevalence of and associated social factors with suicidal behaviors in the Mediterranean countries.

Keeping the relaxed Mediterranean life style in mind one may anticipate lower suicide rates in the region. The findings obtained from this analysis confirmed this anticipation. The age standardized suicide rates in the Mediterranean countries were indeed lower than the world average. The average suicide rate in the world in 2012 was 11.4 (15.0 for males and 8.0 for females) per 100,000 population. The average suicide mortality rates observed in the Mediterranean region (6.04 for both-sexes; 9.29 for males and 2.95 for females) show clearly that the region has a distinct feature at the world scale. There are only three countries in the region that report above the world average suicide mortality rates: France, Slovenia and Croatia.

One feature of suicidal behavior is that it presents a large intersocietal variation [58, 59]. The results from this analysis have also shown that the rates of suicide mortality vary considerably between the Mediterranean countries. The rates reported in Table **1** reveal some consistent yet contradictory patterns. The results from this study show that both-sex and male age standardized suicide mortality rates are lower in Muslim than in the Judeo-Christian countries but the rates for females are similar. The highest suicide rates are observed in the three largely Catholic nations: Slovenia, France and Croatia. Overall, the Arab Muslim nations report lowest suicide rates in the region. They also report lower suicide rates than the non-Arab Muslim nations: Turkey, Bosnia-Herzegovina and Albania. There is a strong suspicion that Arab nations mis/underreport suicides. For instance, Pritchard and Amanullah have shown that most suicides are misclassified as Other Violent Deaths in Middle Eastern Muslim Arab nations but not in non-Arab European Muslim nations such as Albania, Bosnia-Herzegovina or Turkey [60]. There is a religio-cultural stigma attached to suicide in Muslims. The stigma may jeopardize help-seeking when it is indeed needed most.

As with suicide mortality, the rates of non-fatal suicidal behaviors vary considerably between the countries under scrutiny. According to the findings reported in Table **2**, the highest percentages of suicidal ideation are seen in Turkey, Croatia and Slovenia. The lowest percentages are noted in France, Spain, Lebanon. The highest percentages of suicide attempts are seen in Palestine, Cyprus, Greece and Slovenia. The lowest rates were in Spain, Italy, Tunisia and Lebanon. The literature search about non-fatal suicidal behaviors in the Mediterranean countries revealed that the extant scholarly work on these behaviors is limited. There were no studies about suicidal thoughts and attempts in seven countries. Thus, there is an urgent need for more scientific work on suicide in this region.

Social and contextual factors play a role in the intersocietal variation of suicidal behavior. For the present investigation, the association of six social and contextual factors to suicide rates were examined at bivariate and multivariate levels. The bivariate analyses indicated that human development and democracy indices, percentage of population saying religion is unimportant in daily life and GDP per capita were positively but coefficient of human inequality and gender inequality indices were inversely related to suicide rates. It is interesting to note that percentages of depressed and anxious people in a society were unrelated to suicide rates despite an enormous body of research relating mental health problems to suicide. It is also interesting that as the human development and democracy indices increase so does suicide rates in a society. This condition is probably reflecting the association of sociocultural values and attitudes to suicidal phenomena [61, 62].

A statistical problem with bivariate correlation analysis is that there may be multicollinearity between variables. Multivariate statistical methods are used to overcome this problem. The results from the multiple regression analyses have shown that coefficient of human inequality index was found to be the only independent predictor of both-sex and male suicide rates when the effects of other factors are controlled. As the human inequality in a society increases the suicide rates decreases. This may seem counterintuitive at first glance. It is possible that widened human inequality in a society diverts people’s attention to conditions conducive to inequality rather than to their own personal conditions. This may in turn influence attributional processes. That is, people may attribute their personal problems to external conditions (sources for inequality) under high inequality, but they may attribute their problems to their personal characteristics (personal inadequacies) when inequality is low. Indeed, the research indicates that attributional processes are amenable to external manipulation and play a role in people's mood [63].

The research indicates that religion is possibly protective against suicidal behavior [64]. For instance, religiosity is a protective factor against suicidal mortality [32, 65] and is associated with low suicide acceptability [66]. Some research findings indicate that religious affiliation is not protective against suicidal ideation but protective against suicidal attempts [67] and some research findings show that suicidal affiliation can indeed be a risk factor for suicide ideation in depressed patients [68]. Based on these observations it was anticipated in this study that percentage of people saying religion is unimportant in daily life in a society would be associated with heightened suicide rates. The data confirmed that this anticipation held true only for female suicide rates. As the percentage of people saying religion is unimportant in daily life increases so does the female suicide rates. It seems that level of religiosity is a protective factor against female suicides in the Mediterranean basin.

## CONCLUSION

This study is the first to explore the aspects of suicidal phenomena in the Mediterranean countries from a comparative perspective. There are some limitations of the present study. Although the study shed some important light on the subject matter, the findings reported in this paper should be approached with caution for several reasons. First, the study has undertaken an analysis of data supplied to World Health Organization by the national governments. Suicide is a disapproved and hence a stigmatized event especially in the Muslim countries. Therefore, the official data are prone to under or misreporting. Second, the associations of social indicators to suicide mortality are correlational and hence causality cannot be inferred. Finally, the study has also showed that there is more need for scholarly work on suicidal behavior in countries surrounding the Mediterranean Sea.

## Figures and Tables

**Table 1:** Age standardized suicide rates per 100, 000 population in Mediterranean countries according to World Health Organization (2016).

Country	**Age Standardized Suicide Rates (per 100, 000 population)**			**M:F Ratio**
	*Both Sex*	*Male*	*Female*	
Albania	5.6	7.0	4.3	1.63
Algeria	3.3	4.9	1.8	2.72
Bosnia and Herzegovina	6.4	10.6	2.5	4.24
Croatia	11.5	18.8	5.1	3.69
Cyprus	4.5	7.2	1.9	3.79
Egypt	4.4	7.2	1.7	4.24
France	12.1	17.9	6.5	2.75
Greece	3.8	6.1	1.5	4.07
Israel	5.2	8.2	2.4	3.42
Italy	5.5	8.4	2.6	3.23
Lebanon	3.2	4.2	2.2	1.91
Libya	5.5	8.7	2.3	3.78
Malta	6.5	10.3	2.8	3.68
Monaco	NA	NA	NA	NA
Montenegro	7.9	12.6	3.6	3.50
Morocco	3.1	2.5	3.6	.69
Palestine	NA	NA	NA	NA
Slovenia	13.3	22.4	4.5	4.98
Spain	6.1	9.3	3.1	3.00
Syria	2.4	3.8	1.1	3.45
Tunisia	3.2	4.4	2.2	2.00
Turkey	7.2	11.3	3.2	3.53

**Table 2:** Suicidal ideation and attempts in community samples of youth in the Mediterranean countries.

**Country**	**Study Details**	**S-Ideation**	**S-Attempt**
Albania	No study available	-	-
Algeria	No study available	-	-
Bosnia & Herzegovina	No study available	-	-
Croatia	Data from 45,806 high school students aged 15-16 years from 17 European countries (Croatia n = 3008) [51].	32.2%	8.4%
Cyprus	Data from 45,806 high school students aged 15-16 years from 17 European countries (Greek Cypriot n = 6340) [51].	26.5%	14.9%
Egypt	Data from 8417 university students from 12 countries (Egypt n = 653) [52].	17.5%	7.1%
France	Data from 2003 European School Survey Project on high school students (France n = 13,187) [53].	9.59%	8.88
Greece	Data from 45,806 high school students aged 15-16 years from 17 European countries (Greece n = 3060) [51].	26.4%	12.5%
Israel	Data from 1770 Jewish Israeli 10th-grade pupils [54].	17.4%	7.1%
Italy	Data from 5572 university students from 12 countries (Italy n = 471) [9].	20.5%	2.6%
Lebanon	Data from 8417 university students from 12 countries (Lebanon n = 706) [52].	12.3%	5.1%
Libya	No study available		
Malta	No study available		
Monaco	No study available		
Montenegro	No study available		
Morocco	Self-report survey of suicidal behaviors in a total of 3020 students (53% boys) aged 11-23 years (average age = 16 ± 2.1 years) in the North-Centre region of Morocco among students in public secondary schools [55].	15.7%	6.5%
Palestine	Data from 8417 university students from 12 countries (Palestine n = 793) [52].	23.6%	17.6%
Slovenia	Data from 45,806 high school students aged 15-16 years from 17 European countries (Slovenia n = 3085) [51].	30.8%	12.5%
Spain	Survey of 2118 Spanish university students about 12-months prevalence of suicidal ideation and attempts [56].	9.9%	0.6%
Syria	No study available		
Tunisia	Eskin *et al*. Data from 8417 university students from 12 countries (Tunisia n = 707) [52].	19.2%	5.0%
Turkey	Data from 3031 Turkish university and high school students [57].	36.1%	9.4%

**Table 3:** Bivariate correlation coefficients between social indicators and suicide mortality rates.

**Social Indicator**	**Both Sex Suicides**	**Male Suicides**	**Female Suicides**
Percentage of population depressed	0.295	0.295	0.235
Percentage of population anxious	– 0.065	– 0.113	0.083
Alcohol consumption per capita	0.667*	0.660*	0.544*
Unemployment rate (% unemployed)	– 0.126	– 0.094	– 0.195
Human development index	0.550*	0.549*	0.421**
Coefficient of human inequality	– 0.690*	– 0.717*	– 0.445**
Gender inequality index	– 0.481*	– 0.496*	– 0.310
GDP per capita (PPP)	0.477*	0.492*	0.274
Democracy index	0.434**	0.413**	0.412**
Religion unimportant (%)	0.647*	0.608*	0.640*

* p < 0.05; ** p < 0.10.
